# Acceptance of an upper body exoskeleton for occupational tasks among soldiers

**DOI:** 10.3389/fpubh.2026.1793794

**Published:** 2026-06-18

**Authors:** Tim Schubert, Robert Weidner

**Affiliations:** 1 Laboratory for Manufacturing Technology, Helmut Schmidt University/University of the Federal Armed Forces Hamburg, Hamburg, Germany; 2 TU Bergakademie Freiberg, Freiberg, Germany

**Keywords:** acceptance, ergonomic support, exoskeleton, occupational health, wearable assistive technology

## Abstract

**Introduction:**

This study investigated the user experience and perceived acceptance of a shoulder exoskeleton during Chemical, Biological, Radiological, and Nuclear (CBRN) tasks in a military context. While laboratory research on the same system has shown measurable reductions in muscle activity of 29%–41%, the present field study focused on how end users subjectively evaluate the exoskeleton.

**Methods:**

Twenty-seven soldiers of the German Armed Forces tested the exoskeleton for approximately 30 min while completing standardized scenarios, including overhead lifting, vehicle decontamination with a spray lance, and casualty rescue. Immediately afterward, participants completed a structured questionnaire assessing usability, wearing comfort, impact on work routine, perceived support, usefulness, skepticism, and intention to use.

**Results:**

Overall, the exoskeleton was rated as easy to operate (mean item score: 5.15 ± 1.31 on a 7-point scale) and reasonably comfortable to wear (4.64 ± 1.31). Compatibility with work routines and perceived usefulness received moderate ratings (3.79 ± 1.53 and 3.05 ± 1.64, respectively), whereas the perceived mechanical support was notably low (2.78 ± 1.78). Skepticism (2.27 ± 1.08), and intentions to use the system were moderate (3.42 ± 2.08).

**Discussion:**

The user feedback suggests that while the current design is usable and generally comfortable, acceptance may improve substantially if future systems provide a higher amount of support and an expanded functionality to address a wider variety of tasks. These findings underscore that, beyond biomechanical effectiveness, user acceptance is a critical factor for the successful implementation of exoskeletons as occupational health interventions.

## 1 Introduction

Soldiers operating in chemical, biological, radiological, and nuclear (CBRN) environments are exposed to physically demanding tasks that place significant strain on the musculoskeletal and cardiovascular systems (1). Activities such as manual decontamination of vehicles and equipment using spray lances, handling of heavy loads or performing lifting tasks and overhead tool use are typically carried out while wearing protective suits and respiratory systems, which further increase thermal and mechanical load, restrict mobility, and exacerbate fatigue. As a result, CBRN personnel face an elevated risk of work-related musculoskeletal disorders including reduced performance, fitness and health (2).

In recent years, wearable robotic systems such as exoskeletons have emerged as a promising approach to mitigate these physical demands. Exoskeletons are designed to reduce the biomechanical loads on specific joints or muscle groups by supporting or redistributing external forces during task execution (3). While such technologies have been increasingly explored in industrial settings, their applicability to military scenarios remains less understood (4). Compared to civilian workplaces, CBRN operations impose unique challenges due to the protective gear and suits, which restrict mobility and cause thermal discomfort and skin moisture (5). Therefore, any wearable support system needs to be lightweight, unobtrusive, and fully compatible with existing mission gear.

Existing industrial exoskeletons are often optimized for controlled environments, which typically do not need to maintain airtight barriers between the user and a contaminated environment. Consequently, their direct deployment in CBRN operations is not feasible without substantial modification. To address these challenges, a pneumatically actuated shoulder exoskeleton was recently developed and evaluated under laboratory conditions. The system was designed to maintain compatibility with CBRN protective equipment, integrate sealed pneumatic and electrical ports, and provide adjustable support for upper-limb tasks. Laboratory findings demonstrated that the system substantially reduced muscle activity in the anterior deltoid up to 41%, lowered cardiovascular strain, and enabled improved arm posture depending of the specific task (6). However, laboratory assessments alone cannot fully capture the complex interactions between the user, the system, and the operational environment.

A key reason for the still limited widespread adoption of exoskeletons is the lack of clear evidence regarding their effectiveness in actual workplace environments (use case independent). Although manufacturers can assess load-reducing capabilities under controlled laboratory conditions, the real performance of an exoskeleton is highly use-case specific and depends on the task, environment, and experience of the user. Laboratory studies mainly validate biomechanical effects, quantify reductions in physical effort, and identify potential undesired load redistributions. However, such investigations rely on equipment such as motion-capture systems, force platforms, or electromyography (EMG) that cannot always be deployed in the field (7). Moreover, comparative studies have shown that muscle activity measured in the field during manual material-handling tasks differs significantly from that obtained under laboratory conditions (8). In contrast, field studies are required to determine whether an exoskeleton provides meaningful benefits in real work settings, including its influence on perceived exertion, usability, acceptance, and discomfort. Conducting evaluations at real-life workstations also enables experienced users to provide feedback on practical issues that may only emerge during operational use, thereby offering insights about the acceptance of the technology from end-users, that cannot be captured in controlled laboratory environments (9).

Technology acceptance is a multidimensional construct and can be understood as an individual decision-making process that ultimately leads to the voluntary use of a new system. In the context of human–technology interaction, it is commonly defined as the willingness of a user group to employ a technology for the tasks it is intended to support (10). Research in this field aims to identify the determinants that influence users’ intention to use a system and to derive guidelines for designing technologies that are more accepted. Various theoretical models have been proposed to explain how such influencing variables interact. Among these, the Technology Acceptance Model (TAM) is one of the most widely applied frameworks. Besides various technology specific factors, it emphasizes perceived usefulness and perceived usability as central predictors of behavioral intention and actual system use. According to this view, users adopt a technology primarily when they expect performance benefits and when the effort required for its operation is low (11). While TAM focuses on intention formation and the pragmatic attributes of a system, the Technology Usage Inventory (TUI) is based on the TAM and provides an extended perspective that is particularly suitable for evaluating early-stage prototypes (12). Unlike TAM, which was originally developed for software applications, the TUI integrates additional psychological determinants such as emotional responses, anxiety, skepticism, and interest to measure the intention to use. Therefore, it allows a more comprehensive assessment of how individuals perceive and evaluate new assistive devices into their work routines.

Although models such as TAM and its derivatives are widely applied, they have also been criticized for their limited ability to predict actual system use. Prior reviews show that perceived usefulness and ease of use (usability) are weaker predictors of usage than behavioral intention, and that many acceptance frameworks employ overlapping or insufficiently defined constructs (13). Because these models were originally developed for information systems and have since been adapted inconsistently across domains, there is no clear consensus on which factors most reliably explain acceptance of emerging technologies. A recent review by Elprama et al. (14) identified five overarching categories that influence both intention to use and actual use of occupational exoskeletons. Physiological factors comprise aspects such as users’ prior musculoskeletal complaints and the physical comfort of wearing the device. Psycho-social factors include perceived usefulness, expectations, and previous knowledge about exoskeletons. Work-related factors capture the compatibility between a device and the concrete tasks it is intended to support, while policy-related factors refer to organizational conditions such as mandatory use policies, prevention strategies or shared versus personal allocation of devices. Finally, implementation-related factors concern the ease of using the system in practice, including donning, doffing and operational handling.

Empirical evidence from various application domains shows that acceptance depends strongly on the specific work context and on user’s experiences during real tasks. For instance, field studies with plasterers demonstrated that perceived load reduction, task fit, and a balance of advantages over disadvantages were crucial for continued use of an exoskeleton over several weeks (15). Research on lower-limb industrial robots has similarly shown that usability dimensions such as mobility, adjustability, handling, and perceived safety shape users evaluations and guide further system development (16). In rehabilitation settings, therapists reported that their willingness to adopt upper-limb exoskeletons was primarily influenced by perceived usefulness and prior experience with robotic systems (17). Studies involving older adults highlight that acceptance is shaped by emotional factors, potential embarrassment, and concerns about stigma, emphasizing the need for user-centered design (18). The agricultural sector further illustrates that insufficient acceptance is often linked to a mismatch between system capabilities and real environmental constraints, underscoring the relevance of participatory design and context-specific adaptation (19). Additional research in automotive and industrial contexts indicates that intention to use is strongly tied to usability and wearing comfort (20), and that perceived usefulness and ease of use are key determinants for early adoption (14). More recent studies also demonstrate that self-perceived attractiveness and social feedback from colleagues can shape acceptance over time, especially during initial use phases (21, 22).

To date, field-based research on shoulder exoskeletons in military settings remains limited. Therefore, the aim of the present study was to evaluate the developed shoulder exoskeleton under realistic field conditions with operational end-users of the German armed forces. In addition to the biomechanical benefits demonstrated in laboratory experiments, this study investigated user acceptance in terms of perceived usefulness, usability, intention to use, support, wearing comfort and skepticism. A standardized questionnaire based on dimensions of the Technology Usage Inventory (TUI) was employed to systematically capture subjective user experience after performing typical CBRN-related tasks.

## 2 Method

The following methods section outlines the design of the shoulder exoskeleton and summarizes key findings from the preceding laboratory study, before detailing the field study design, participant characteristics, and the measurement approach, including the applied items and dimensions of the questionnaire.

### 2.1 Design and biomechanical effects of the exoskeleton for shoulder support

**Figure 1:**
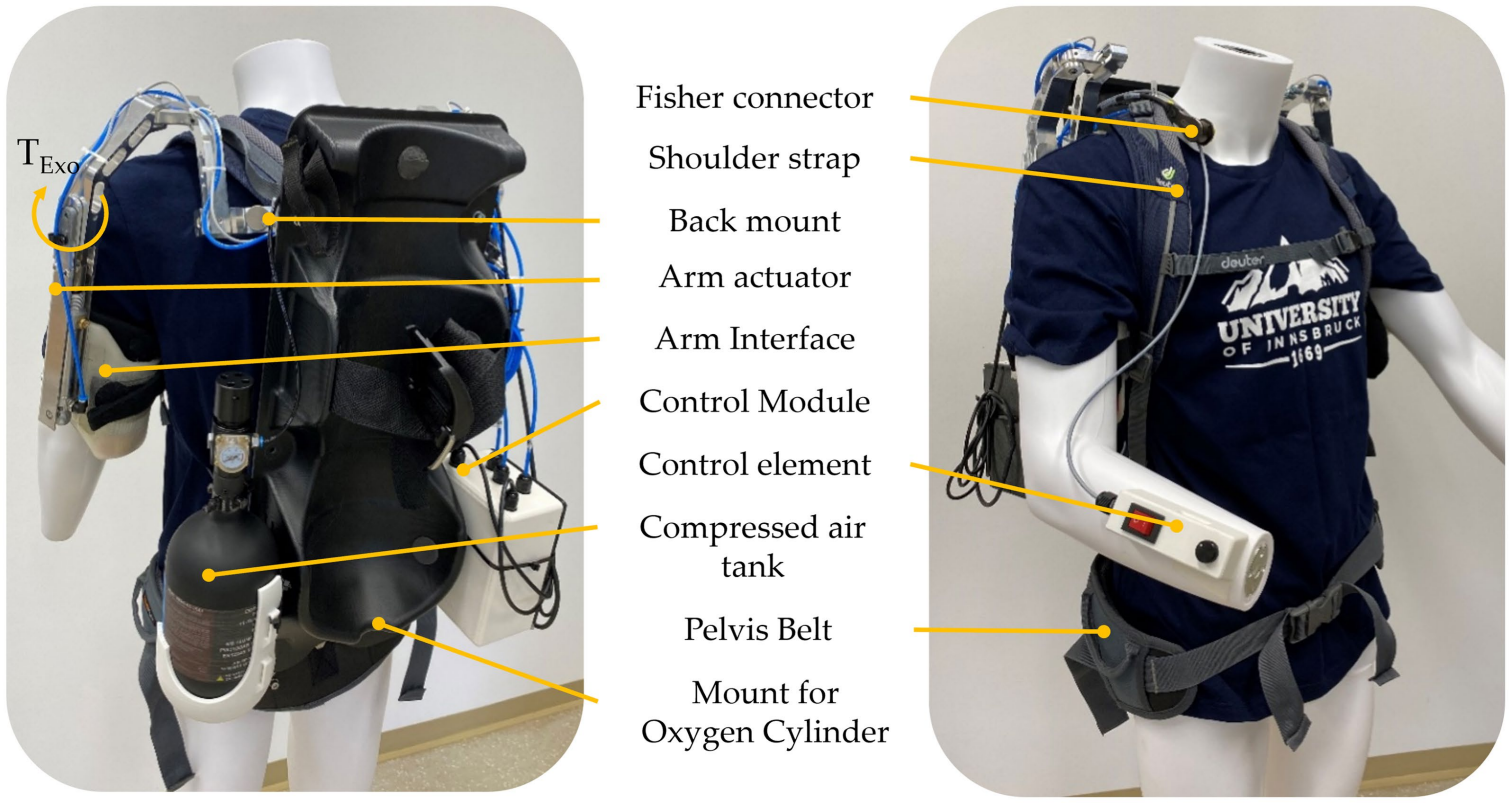
Exoskeleton for shoulder support rear (left) and front view (right) (6).

The shoulder exoskeleton evaluated in this field study is an active, pneumatically actuated support system designed to reduce shoulder load during overhead lifting tasks (see Figure 1), and based on the system Lucy (23, 24). Its mechanical and control architecture has been described in detail in a previous publication (6). In brief, the system consists of a lightweight back structure with an integrated shoulder mechanism providing one actively actuated degree of freedom (shoulder flexion/extension) and two passive degrees of freedom for each arm (left and right to preserve natural movement). The actuation is realized through a compact, integrated pneumatic cylinder generating up to approximately 8 Nm (stepless adjustable) of assistance torque per shoulder at 5 bar system pressure.

A control module mounted laterally on the back frame houses the microcontroller, solenoid valves, and an inertial measurement unit (IMU). Based on sensor input from a rotary potentiometer and the IMU, the controller regulates internal pressure following a predefined parabolic torque curve that corresponds to the biomechanical loading of the shoulder joint. The user can manually adjust the overall assistance intensity via a small wrist-mounted interface.

To ensure compatibility with CBRN protective suits, the system integrates sealed pneumatic and electrical feedthroughs and mounts for externally accessible components, such as the compressed air tank (1.1 L, 300 bar) and power supply unit. The total system weight is approximately 6.5 kg, including all mounted components, and the configuration enables approximately 1,900 actuation cycles, corresponding to about 4 hours of continuous operation in field conditions.

The arm interface consists of a semi-open 3D-printed arm shell that distributes the support force comfortably over the upper arm and can be adapted to different anthropometric sizes. The exoskeleton’s backpack-like design ensures a balanced load transfer from the shoulders to the pelvis via an adjustable back plate and padded shoulder and pelvic straps, providing both stability and comfort during prolonged wear.

Before conducting the field study, the shoulder-support exoskeleton was examined under controlled laboratory conditions to quantify its biomechanical and physiological effects. The experimental evaluation aimed to assess (1) muscular unloading in m. deltoideus anterior addressed by the exoskeleton (via electromyography, EMG), (2) global physiological load and potential relief (via spiroergometry), (3) alterations in movement trajectories during task execution (via motion capture), and (4) subjective perception of exertion and system-induced relief (via questionnaire).

A within-subject design with paired samples was applied, in which all participants performed identical task sequences under two conditions: (1) without exoskeleton (control) and (2) with the shoulder exoskeleton (intervention, 100% assistance). This crossover approach enabled direct comparison between supported and unsupported task execution while minimizing inter-individual variability.

Two representative work tasks were analyzed: an intermittent load task involving the lifting of a 5 kg container from hip to head height, and a continuous load task simulating decontamination by spraying along horizontal and vertical trajectories with a spray lance.

The results showed that during dynamic lifting tasks, the exoskeleton reduced the activation of the m. anterior deltoid by up to 29%, while enabling comparable or slightly higher arm elevation angles. In static or semi-static tasks, such as horizontal spraying, muscular activity decreased by approximately 41%, indicating that the system provided effective load compensation throughout sustained overhead work. Vertical spraying movements showed similar benefits, with muscle activity reduced by around 37%.

In addition to the muscular effects, subjective ratings of perceived exertion (RPE) decreased by 31% during lifting and by 38% during spraying, confirming the objective results. Cardiovascular load, measured by heart rate and oxygen consumption, also declined significantly across both tasks, suggesting an overall reduction in physical workload.

These findings confirm that the shoulder exoskeleton can effectively reduce muscular and physiological strain during overhead and forward-reaching activities. Based on these results under laboratory conditions, a subsequent field study was conducted to assess user acceptance, comfort, and practical applicability during real-world scenarios.

### 2.2 Participants and study design

**Figure 2:**
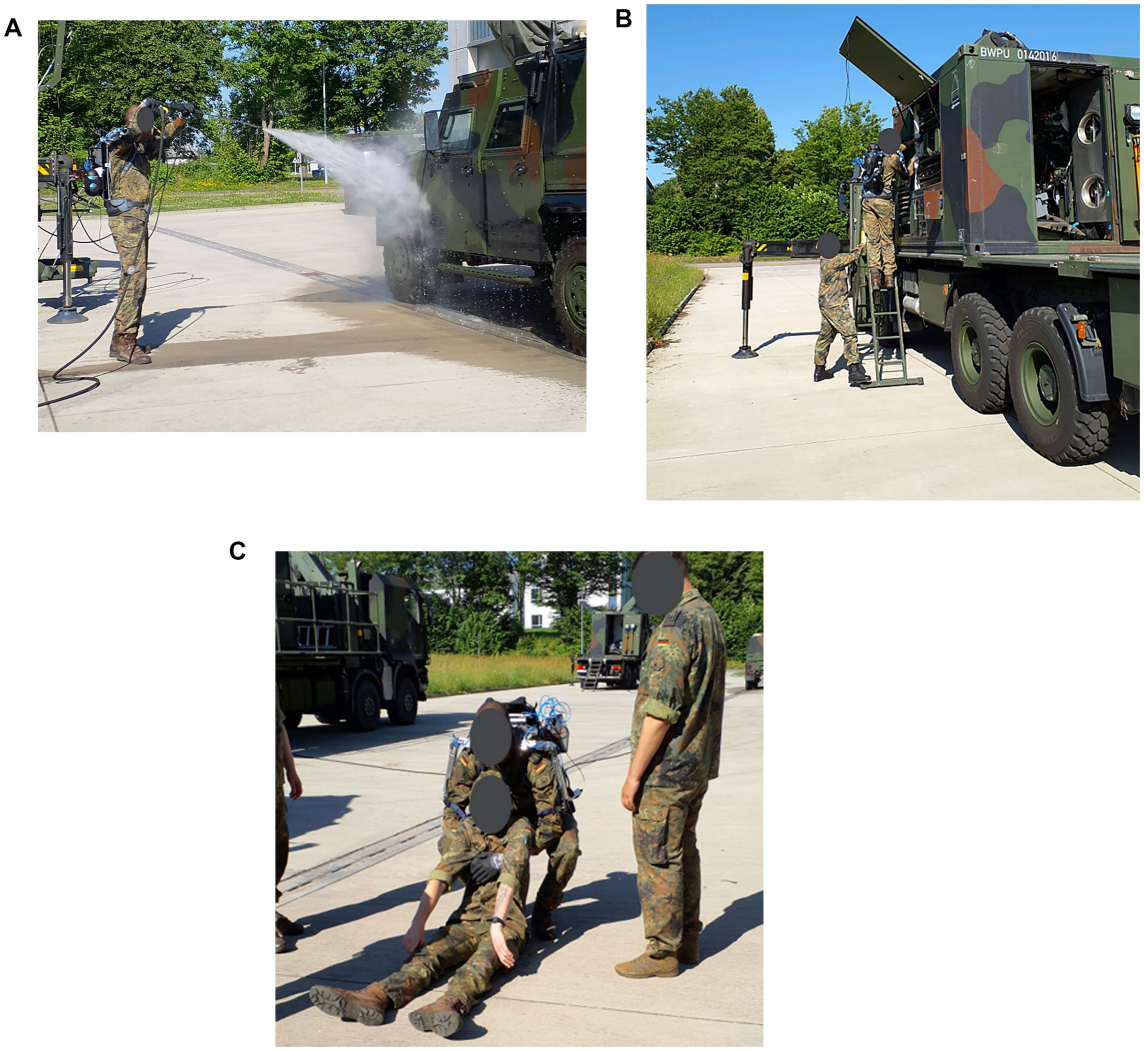
Workstations for exoskeleton use: **(A)** decontamination using a spray lance, **(B)** lifting objects into vehicle, **(C)** rescue of persons.

A field study was conducted to evaluate the exoskeleton through a standardized questionnaire survey administered to end users of the German Armed Forces. The sample size was constrained by the limited availability of trained military personnel eligible for participation during the scheduled field exercise. As the study was integrated into an operationally realistic training environment, recruitment opportunities were restricted by organizational and logistical conditions. Therefore, the shoulder exoskeleton was tested by 27 soldiers (23 males, 4 females, 31.2 ± 7.4 years) of the ABC defense battalion 750 in Bruchsal, Germany. Each participant used the system for approximately 30 min under realistic operational conditions. Purpose-designed workstations were set up to simulate relevant tasks, including lifting objects above head-high, decontaminating vehicles using a spray lance and rescue of persons (Figure 2). Immediately after the trials, participants completed a questionnaire designed to capture their subjective perceptions of the systems.

The primary objective of this investigation was to gather insights into the handling, wearing comfort, operational suitability, and user acceptance of the developed technology demonstrators, as well as to identify potential areas for improvement from the perspective of future end users.

An *a priori* power analysis (G*Power 3.1) indicated that detecting a medium-sized correlation (*r* = 0.30) with *α* = 0.05 and power = 0.80 would require *N* = 84 participants. The present sample (*N* = 27) is therefore primarily sensitive to large effects (*r* ≈ 0.50). Consequently, the correlation analyses should be interpreted as exploratory.

### 2.3 Measures

The questionnaire used in this study was based on the Technology Usage Inventory (TUI), a validated instrument designed to assess user acceptance and attitudes towards new technologies (12). Responses were provided using a 7-point Likert scale, ranging from 1 (strongly disagree), through 4 (“neither agree nor disagree”), to 7 (strongly agree). Although the TUI consists of multiple dimensions, this field study focused on the four key dimensions that were most relevant to the evaluation of the exoskeleton in a military context: intention to use, usability, perceived usefulness, and skepticism. Other TUI dimensions such as curiosity, accessibility and immersion were not included because they were considered less relevant for the practical field evaluation focus of the present study. The original TUI items were linguistically and contextually adapted to match the specific characteristics of the exoskeleton and the operational scenarios in which they were tested. For example, within the dimension intention to use, the original TUI item “Would you use this technology?” was adapted to “I would use the exoskeleton during my work.” Similarly, within the dimension usefulness, the original item “The use of the technology would make many tasks more comfortable” was adapted to “The use of the exoskeleton would make many tasks more comfortable.”

The dimensions including the items are explained in more detail below:

*Intention to use*: the first three questionnaire items addressed the participants’ intention to use the exoskeletons. Intention to use reflects the willingness and interest of potential users to adopt the technology in their daily work environment, thereby representing a central predictor of acceptance and long-term integration. Three specific questions were formulated: (1) general willingness to use the exoskeleton during work, (2) desire for acquisition of the system by the organization, and (3) personal interest in having access to the system during work.

*Usability*: the next category focused on usability, referring to how easily and intuitively the exoskeletons can be operated in practical work situations. Three items were included: (1) intuitive and effortless handling during use, (2) ease and comfort of donning and doffing, and (3) a negatively phrased item addressing perceived complexity of operation. A low score on the third item indicated high usability.

*Perceived usefulness*: perceived usefulness was assessed through four items examining the extent to which the systems were perceived to facilitate daily work tasks, increase efficiency, and improve comfort. The questions addressed (1) perceived increase in comfort compared to working without assistance, (2) perceived reduction in physical workload, (3) perceived value of the system to the extent that users would consider purchasing it privately, and (4) overall perceived functional benefit of the system for daily tasks.

*Skepticism*: skepticism refers to reservations or concerns users may have regarding the use of the system. Four items were used: (1) perceived risks associated with use, (2) perceived safety hazards or misuse risks, (3) perceived potential disruption to daily routines, and (4) general assessment of advantages versus disadvantages of exoskeleton use.

In addition to the TUI-based categories, three supplementary categories were included to inform further development efforts:

*Wearing comfort*: three questions assessed physical comfort, including freedom of movement, perceived weight, and overall wearing comfort. Three items were used to assess this dimension: (1) perceived restrictions in freedom of movement while wearing the system, (2) the appropriateness of the system’s weight during task execution, and (3) the overall comfort experienced when wearing the exoskeleton.

*Impact on work routine*: three items evaluated how the systems might affect the participants’ daily work routine, both positively and negatively. These items addressed whether the exoskeletons (1) increased enjoyment of work, (2) improved accuracy and effectiveness and (3) enhanced efficiency or saved time.

*Social perception*: one single-item measure assessed perceived social perception related to the visible appearance of the exoskeleton.

*Perceived support level*: a final single item assessed whether the level of assistance provided by the systems was perceived as sufficient for the respective tasks.

*Criticism*: additionally, one open-ended question was included to collect qualitative feedback about what was most disturbing about the exoskeleton.

The specific questions within the dimensions are listed in Table 1.

**Table 1:** Dimensions and items of the questionnaire.

Dimension	Items
Intension to use (TUI)	I would use the exoskeleton during my work. I would like the exoskeleton to be purchased. I would like to have access to this exoskeleton.
Usability (TUI)	The exoskeleton is easy to use. The exoskeleton is easy to put on. The use of the exoskeleton is complicated.
Perceived Usefulness (TUI)	The use of the exoskeleton would make many tasks more comfortable. The exoskeleton makes my work easier. If I could afford it, I would buy the exoskeleton. The exoskeleton would support me in my daily work tasks.
Skepticism (TUI)	I think the use of an exoskeleton is always associated with a certain risk. I think the exoskeleton involves dangers. The exoskeleton would disrupt my daily routine. The use of the exoskeleton would bring more disadvantages than advantages.
Impact on work routine	Working with the exoskeleton is more enjoyable. Working with the exoskeleton improves accuracy/effectiveness. The exoskeleton helps me complete my work faster.
Wearing comfort	My freedom of movement was restricted by the exoskeleton. The weight of the exoskeleton is appropriate. The exoskeleton is comfortable to wear.
Social perception	I feel silly when wearing the exoskeleton.
Perceived support level	The support provided by the exoskeleton was sufficient.
Criticism (open question)	What bothered you the most about the exoskeleton?

### 2.4 Data analysis

Descriptive statistics, including mean sum scores, mean item scores, and standard deviations, were calculated for all questionnaire dimensions. Mean item scores were derived by dividing the summed scale scores by the respective number of items to allow comparability across with differing item counts. Normality of the variables was assessed using the Shapiro–Wilk test. Since the assumption of normal distribution could not be met for several variables, non-parametric methods were applied for subsequent analyses. Accordingly, Spearman’s rank correlation coefficients were computed based on the mean item scores to examine associations between the key constructs of perceived usefulness, usability, skepticism, intention to use, wearing comfort, support, and impact on work routine. Statistical significance was evaluated using two-tailed tests with an alpha level of *p* < 0.05. Descriptive subgroup analyses for male and female participants were additionally performed for exploratory purposes. Data preprocessing was performed in Matlab (version R2024a), while the statistical analyses were conducted using IBM SPSS Statistics (version 29.0.1.0).

Open-ended responses were analyzed using an inductive qualitative content analysis approach. Due to the brevity of responses (typically 1–2 sentences per participant), the analysis focused on descriptive categorization rather than in-depth interpretative thematic development. All responses were read repeatedly by the primary researcher to identify recurring meaning units and patterns in the participants’ feedback. Initial codes were assigned to statements with similar content and subsequently grouped into broader categories reflecting shared themes, such as limited range of motion, insufficient support activation, or wearing discomfort. For example, statements such as “I couldn’t raise my arms fully” and “Arm rotation was restricted” were initially coded as restricted arm mobility and later combined within the category “Limited range of motion”. The preliminary categorization was reviewed and discussed within the research team, and categories were iteratively refined until consensus regarding their content and naming was reached. Due to the small dataset size, no specialized qualitative analysis software was used. The resulting categories represent recurring patterns in participants’ responses and were used to complement the quantitative findings rather than to derive statistically generalizable conclusions.

For each questionnaire dimension, internal consistency was assessed using McDonald’s omega. Negatively worded items (item 6, 18, 21) were reverse-coded prior to the analysis to ensure that higher scores consistently reflected more positive evaluations. McDonald’s omega indicated good to excellent internal consistency for the dimensions “intention to use”, “usefulness”, “work routine” and “usability” (*ω* = 0.83–0.95). For “skepticism” (*ω* = 0.73) and “wearing comfort” (*ω* = 0.71) internal consistency was acceptable for the shoulder exoskeleton. The dimensions “support” and “social perception” consisted of fewer than three items, therefor no omega was calculated. Corrected item-total correlations ranged from 0.34 to 0.91, indicating acceptable to excellent associations between individual items and their corresponding scales. The lowest value was observed for one item within the usefulness dimension, whereas most items showed moderate to high correlations (see Appendix 1).

## 3 Results

The results section presents the descriptive findings of the questionnaire dimensions, the correlations between the different dimensions, and the categorized feedback derived from participants’ open-ended responses regarding limitations of the shoulder exoskeleton.

### 3.1 Descriptive results

As shown in Table 2, the field study captured participants’ subjective evaluation of the active shoulder exoskeleton for CBRN scenarios across seven dimensions. To ensure comparability across dimensions with differing item counts, results are reported as mean item scores on the original 7-point Likert scale, with higher values indicating stronger agreement with the respective dimension.

**Table 2:** Descriptive results of questionnaire.

Dimension	N	Number of items (7-point scale)	Mean sum-score (SD)	Mean item-score (SD)		
				All	Men (*n* = 23)	Woman (*n* = 4)
Usefulness (TUI)	27	4	12.19 (6.55)	3.05 (1.64)	2.97 (1.65)	3.50 (1.72)
Usability (TUI)	27	3	15.44 (3.94)	5.15 (1.31)	5.14 (1.31)	5.17 (1.50)
Skepticism (TUI)	27	4	9.07 (4.30)	2.27 (1.08)	2.10 (0.97)	3.25 (1.27)
Intention to use (TUI)	27	3	10.26 (6.24)	3.42 (2.08)	3.26 (2.04)	4.33 (2.39)
Work routine	27	3	9.33 (4.88)	3.11 (1.63)	3.04 (1.61)	3.50 (1.93)
Wearing comfort	27	3	13.93 (3.92)	4.64 (1.31)	4.70 (1.41)	4.33 (0.27)
Social perception	27	1	5.81 (1.92)	5.81 (1.92)	5.91 (1.90)	5.25 (2.22)
Support	27	1	2.78 (1.78)	2.78 (1.78)	2.57 (1.83)	4.00 (0.82)

Participants generally perceived the shoulder exoskeleton as relatively easy to use, with Usability averaging 15.44 ± 3.94, corresponding to a mean item score of 5.15 ± 1.31 on the 7-point scale. The device was also rated as moderately compatible with participants’ work tasks, with Impact on Work Routine reaching a mean of 9.33 ± 4.88, corresponding to a mean item score of 3.11 ± 1.63. In contrast, the perceived assistance was limited, as indicated by the Support score of 2.78 ± 1.78 on the 7-point scale. Participants expressed moderate skepticism, with Skepticism averaging 9.07 ± 4.30, corresponding to a mean item score of 2.27 ± 1.08, and reported cautious adoption intentions, with Intention to Use at 10.26 ± 6.24 (3.42 ± 2.08 on 7-point scale). Usefulness was similarly moderate, with a mean of 12.19 ± 6.55 (3.05 ± 1.64 on 7-point scale), while Wearing Comfort was perceived more positively at 13.93 ± 3.92 (4.64 ± 1.31 on 7-point scale). Social Perception yielded a mean of 5.81 ± 1.91 on the 7-point scale.

Descriptive subgroup analysis indicated some differences between male and female participants, particularly regarding perceived support, skepticism, and intention to use. Female participants reported slightly higher perceived support (4.00 ± 0.82 vs. 2.57 ± 1.83) and intention to use (4.33 ± 2.39 vs. 3.26 ± 2.04), while also expressing greater skepticism (3.25 ± 1.27 vs. 2.10 ± 0.97) toward the system. In contrast, usability, work routine, social perception and wearing comfort ratings were largely comparable between groups. Given the small number of female participants (*n* = 4), these results are reported for descriptive purposes only.

The standard deviations reveal considerable variability among participants, particularly for Work Routine, Intention to Use, and Usefulness, indicating that experiences with the exoskeleton differed substantially between individuals, whereas Usability and Wearing Comfort exhibited lower variability.

**Figure 3:**
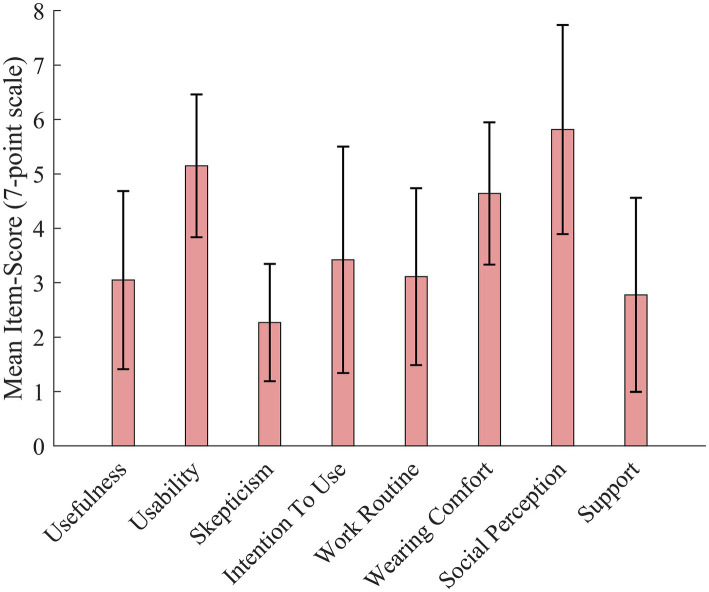
Comparison of mean item-scores per dimension.

In addition to the numerical values presented in Table 2, Figure 3 illustrates the mean item scores on the standardized 7-point scale, providing a visual overview of the relative magnitude of each dimension.

### 3.2 Correlations of acceptance dimensions

Table 3 presents the Spearman correlation coefficients between the evaluated dimensions. Perceived usefulness showed strong positive correlations with intention to use (*ρ* = 0.88, 95% CI [0.75, 0.95], *p* < 0.001), impact on work routine (*ρ* = 0.84, 95% CI [0.67, 0.93], *p* < 0.001), and perceived support (*ρ* = 0.64, 95% CI [0.34, 0.83], *p* < 0.001). Wearing comfort was also positively associated with usefulness (*ρ* = 0.51, 95% CI [0.15, 0.75], *p* = 0.006) and intention to use (*ρ* = 0.49, 95% CI [0.12, 0.74], *p* = 0.009). In contrast, skepticism correlated negatively with usefulness (*ρ* = −0.49, 95% CI [−0.74, −0.12], *p* = 0.010), intention to use (*ρ* = −0.48, 95% CI [−0.74, −0.12], *p* = 0.011), and perceived fit within the work routine (*ρ* = −0.54, 95% CI [−0.77, −0.19], *p* = 0.003). Social perception showed significant positive correlations with work routine (*ρ* = 0.50, 95% CI [0.17, 0.75], *p* = 0.008), wearing comfort (*ρ* = 0.59, 95% CI [0.25, 0.79], *p* = 0.001), and skepticism (*ρ* = −0.46, 95% CI [−0.73, 0.11], *p* = 0.012). Usability showed only weak to moderate and mostly non-significant correlations with the other dimensions. Given the limited sample size, non-significant correlations should be interpreted cautiously, as the study was primarily powered to detect large effects.

**Table 3:** Correlation matrix shoulder exoskeleton.

Variable	M (SD)	Usefulness	Usability	Skepticism	Intention to use	Work routine	Comfort	Social perception	Support
Usefulness	3.05 (1.64)	1.00							
Usability	5.15 (1.31)	0.32	1.00						
Skepticism	2.27 (1.08)	−0.49**	−0.23	1.00					
Intention to use	3.42 (2.08)	0.88***	0.35	−0.48*	1.00				
Work routine	3.11 (1.63)	0.84***	0.35	−0.54**	0.85***	1.00			
Wearing comfort	4.64 (1.31)	0.51**	0.23	−0.38*	0.49**	0.43*	1.00		
Social perception	5.81 (1.92)	0.39*	0.02	−0.48*	0.33	0.50**	0.59**	1.00	
Support	2.78 (1.78)	0.64***	0.14	−0.35	0.46*	0.52**	0.36	0.23	1.00

*p < 0.05, **p < 0.01 and ***p < 0.001 (2-tailed).

### 3.3 Open feedback from participants

The open-ended feedback regarding the shoulder exoskeleton revealed several recurring themes, which were categorized and presented in Table 4.

**Table 4:** Open-ended feedback regarding the use of the shoulder exoskeleton in CBRN scenarios.

Category	Description	Example quotation
Limited range of motion	Users occasionally reported restrictions when raising or rotating the arms, describing the system as bulky or restricting movement.	“I could not raise my arms fully.” / “Arm rotation was restricted.” / “It was very bulky and pressed on the shoulders.”
Delayed or insufficient support activation	Participants indicated that the assistive force activated too late, only at or above shoulder height, providing little help during initial lifting phases.	“The support comes too late.” / “Assistance starts only above shoulder level.”
Lack of support for lower arm segments	Several users noted that the exoskeleton provided no assistance for the forearms, which increased the effort required for overhead tool handling.	“The lower arm part is not supported.” / “Support of the forearms would be a good addition.”
Insufficient adjustability and fit	Participants reported difficulties adapting the exoskeleton to individual body dimensions; for smaller users, the frame was too large.	“It cannot be adjusted precisely to the user.” / “For smaller users, it was slightly too big.”
Comfort and contact pressure	Pressure points and discomfort were reported, particularly at the shoulder interface when working overhead or fully extending the arms.	“When working above the shoulders, the frame pressed on my shoulders.”
Mechanical locking and control suggestions	Users suggested an optional mechanical locking or holding function at the elbows to maintain the arms in position, as well as more refined control mechanisms.	“A locking mechanism at the elbows would help hold the tool.” / “The control system is not yet mature.”
Perceived bulkiness and interference	Users mentioned bumping into obstacles due to its width.	“You underestimate the width and get stuck somewhere.” / “It was very bulky and limited movement.”
Functionality	Some participants found the system useful but desired broader functional range.	“Nothing really disturbed me, but it could support more.”

The most frequently mentioned issue was restricted range of motion, particularly when lifting the arms above shoulder height or performing rotational movements. Participants described the system as “bulky” and noted that “the shoulders hit the upper frame” when fully extending the arms. Many users also reported that the assistive support engaged too late, providing noticeable assistance only at or above head level. Several participants suggested that the system should begin supporting earlier, especially during the initial phase of lifting or tool handling. Another common theme concerned the lack of elbow/forearm support, which was seen as a limitation during overhead tasks such as handling the decontamination lance. Comfort and fit were also recurring issues: some users reported shoulder pressure or limited adaptability of the harness system. A number of participants suggested control-related improvements, such as a locking mechanism at the elbows to temporarily hold the arms in position, and a more responsive control of the assistive torque. Lastly, some subjects indicated a tendency to collide with nearby objects due to its width.

## 4 Discussion

This study evaluated user acceptance of a active shoulder exoskeleton for CBRN scenarios in a real-world field setting, showing that the device was perceived as usable and generally comfortable but provided limited functional support and shows variability in perceived usefulness and skepticism. In the following, these findings are discussed with regard to the descriptive results, the correlations, and the qualitative feedback, followed by a consideration of methodological limitations.

### 4.1 Descriptive results

The descriptive findings reveal that users rated the exoskeleton as easy to operate and generally comfortable, yet they expressed only moderate perceptions of usefulness and intention to use, and they assessed the mechanical support as comparatively low. The moderate Skepticism scores further underline that some participants remained cautious about the overall value and reliability of the system.

Participants consistently evaluated the exoskeleton as usable, as reflected by the relatively high mean score and the low variability in usability. This indicates that donning, adjustment, and operation procedures were well understood and did not pose substantial barriers during field use. Wearing comfort also showed relatively positive and stable ratings, suggesting that the physical interface of the device was adequate for most users during typical work tasks, despite individual differences in anthropometry.

In contrast, ratings for usefulness and intention to use were only moderate, probably due to the low rating of support. This subjective assessment stands in contrast to the results of the preceding laboratory study, in which the same device demonstrated substantial biomechanical effectiveness. In controlled conditions, the exoskeleton reduced shoulder muscle activity by approximately 29% to 41% and did not restrict movement. In fact, an increased range of motion in shoulder flexion and abduction was observed. Comparable findings have been reported by Park et al. (25), who demonstrated that subjective satisfaction with a shoulder exoskeleton was not significantly related to reductions in muscle activity, despite objectively measurable EMG decreases. Instead, users’ evaluations were more strongly associated with unfavorable changes in joint kinematics, such as increased shoulder flexion or neck extension.

The discrepancy between objectively measurable support and subjectively perceived assistance suggests that users may not fully recognize or interpret the biomechanical benefits during dynamic and operational tasks. Several factors may account for this divergence. The field setting involved familiar work tasks, but performing these tasks while wearing the exoskeleton was unfamiliar to participants, potentially increasing cognitive demands and limiting their ability to detect subtle improvements in muscular effort (26). The support profile of the device may also have been insufficiently noticeable during specific movements or operational contexts, leading users to judge the assistance as weaker than it biomechanically was. Furthermore, the relatively high standard deviations in work routine, intention to use, and usefulness indicate a high variability regarding the individual experiences, with some users finding the exoskeleton highly supportive and well-integrated into their work, while others reported limited benefit or difficulty in performing overhead tasks. This variability may reflect differences in individual physical characteristics, task familiarity, experience and subjective expectations and could therefore contribute to the observed discrepancy between objective measured support and the subjective perceived relief of the exoskeleton.

Beyond functional and ergonomic aspects, previous research suggests that social dynamics can substantially influence the acceptance of wearable assistive technologies. A recent study demonstrated that both self-perceived attractiveness and social feedback shape users’ intention to continue exoskeleton use, particularly during early adoption phases. Users who received positive reactions from others showed higher willingness to keep using the system, whereas negative comments or nonverbal cues reduced acceptance over time. These effects reflect well-documented psychological mechanisms related to social connectedness, stigma, and impression management (22).

Although the item “I feel silly when wearing the exoskeleton” captures aspects of appearance and perceived social evaluation, its relevance in this study is limited. In contrast to civilian workplaces, social feedback usually plays a smaller role in military settings, where cohesion, equality and adherence to operational requirements dominate and equipment use is a standardized procedure. These conditions reduce the likelihood that individual aesthetic concerns have a meaningful influence on acceptance.

### 4.2 Correlations of shoulder exoskeleton

The correlation analysis supports associations that are consistent with theoretical models of exoskeleton acceptance, in which perceived usefulness is considered a primary factor related to intention to use. In our data, perceived usefulness showed a strong positive association with the intention to use and the impact on daily work routine. This suggests that users may be more willing to adopt an exoskeleton when they believe it offers real functional benefits in their task environment, a relation that has also been reported in field studies across diverse application domains, including the industrial automotive sector (27), logistics (28), in rehabilitation settings (17), and care work (29). Comparable findings have also been observed in military contexts, for example in a recent field evaluation with U.S. Army soldiers, where the majority reported enhanced performance and a high likelihood of future use of the device (30).

The significant correlation between work routine compatibility and the other variables (except usability) indicates that users responded more positively in terms of usefulness and intention to use, when the exoskeleton fits well with the demands of their daily tasks and has a positive impact on to their work. This finding aligns with Schwerha et al. (31), who identified major factors contributing to exoskeleton use intention as perceived comfort, task-technology fit, perceived safety, and perceived usefulness. Their results emphasize that a good fit between the device and daily work tasks plays a crucial role in creating a positive user acceptance and adoption. Research on military exoskeletons provides comparable evidence. Mudie et al. (32) emphasize that successful exoskeleton use requires alignment with the user, the task and the environment. Because many military systems do not meet these requirements, they fail to support daily work routines and therefore have not yet been adopted for operational use.

Skepticism, on the other hand, emerged as a significant barrier, showing negative relationships with usefulness, intention to use, wearing comfort, social perception and impact on work routine. This suggests that doubts about safety or practical and social disadvantages may hinder adoption. Physical aspects such as wearing comfort and perceived support were moderately related to usefulness and intention to use, emphasizing that ergonomic optimization and clear functional benefit are both crucial for acceptance. Interestingly, Usability seems less important, as it shows only weak to moderate associations with intention to use, indicating that users may tolerate minor handling issues as long as the exoskeleton provides tangible support during physically demanding tasks. However, these non-significant relationships should be interpreted with caution given the limited statistical sensitivity of the present sample.

### 4.3 User feedback – open questions to shoulder exoskeleton

The open feedback provided further insight into how participants perceived the functionality and practical relevance of the shoulder exoskeleton in their daily work environment. A frequently raised point in the participant interviews concerned limitations in the range of motion. Several participants tested the maximal arm elevation during the familiarization phase and deliberately stretched their arms far above shoulder height. Such extreme movements were not observed during the work tasks performed with the exoskeleton and therefore do not reflect typical task demands. The perceived restriction may thus partly stem from exploratory testing rather than from limitations experienced during actual task execution. Participants also described the system as bulky. This feedback is consistent with evidence that inadequate adjustability is a common challenge, particularly for petite or smaller-bodied users who are more affected by the weight and geometry of an exoskeleton when the fit cannot be sufficiently adapted to their anthropometry (33). Although the evaluated system provides several configurational options, including adjustable back-bar length, shoulder structure, and size-adaptable arm and hip straps, these mechanisms appear to be insufficient for a subset of users. Prior research has shown that poor adjustability and an inadequate fit can negatively influence comfort, with discomfort and localized pain acting as critical determinants of the subjective fit experience (34).

Participants also noted that the activation of the assistance occurred too late during arm elevation. The control system was configured to initiate support at approximately 35 degrees of shoulder elevation to avoid unintended lifting forces in the lower range of motion where assistance is typically not required. However, this threshold appears to have been too low for several real work tasks. Determining an appropriate activation angle requires a detailed analysis of typical arm postures across a wide range of tasks in the user’s occupational environment in order to identify the point at which shoulder loading becomes meaningful. Future developments could address this limitation by implementing adaptive control strategies, including artificial intelligence based algorithms that continuously learn from user movements and automatically adjust activation thresholds to individual work patterns.

As already observed in the descriptive results, the perceived assistance provided by the exoskeleton was rated as insufficient by many users. This perception may partly stem from a mismatch between user expectations and the actual function of the device. Participants might underestimate the support since the tasks remain physically demanding in the short term, leading to a feeling that the exoskeleton provides limited relief. However, the primary benefit of an exoskeleton lies in its potential to reduce cumulative musculoskeletal load over prolonged use, which is not immediately apparent during brief testing sessions. Moreover, the user feedback and the objective findings on strain reduction reveal a clear discrepancy between the level of support users desire from the system and the amount of relief actually required to effectively reduce musculoskeletal risks. To the best of our knowledge, there is no defined threshold on EMG reductions that defines sufficient exoskeletal assistance for both injury prevention and user satisfaction. While many studies report objective reductions in muscle activity in the range of 15% to 40% (35–37), there is currently no validated threshold that specifies how much EMG reduction is necessary to effectively lower the risk of musculoskeletal disorders. Moreover, subjective perceptions of assistance do not reliably map onto EMG reductions. This mismatch is also reflected in the findings of Zindashti et al. (38), who reported that muscle activity reflects perceived (subjective) support only to a limited extent, with an average similarity index of 49% (*r* = 0.27). Their results indicate that users often sense the provided relief only partially, even when measurable reductions in muscle activity are present. Consequently, although our laboratory reductions of 29%–41% are within the range commonly reported, we cannot assume that this magnitude alone explains user satisfaction or guarantees reduced long-term injury risk.

In addition to users’ desire for increasing the level of support, a recurring topic was the wish for a broader range of assistance from the exoskeleton. Users often felt the device supported only a narrow set of motions, mainly the lifting of the arms. Many participants requested a broader range of functional benefits that would also assist the elbow, back or lower body during common tasks such as lifting objects from the ground. From their perspective, the limited versatility reduced the overall utility of the system, particularly when considering the time and effort required to put the device on. This limitation is common among existing exoskeletons. As noted by Tian et al. (39), most exoskeletons provide single-joint support, which limits their applicability in tasks requiring multi-joint coordination. Therefore, acceptance could be improved if future systems integrate assistance for multiple joints, thereby enabling a broader range of applications.

### 4.4 Limitations

Although the field study provides valuable first insights into the acceptance of the shoulder exoskeleton, several limitations must be considered when evaluating the findings. First, the results must be interpreted regarding a very short exposure time. Acceptance of exoskeletons is known to develop dynamically over prolonged periods of use, during which initial impressions may gradually evolve into more stable assessments. Early reactions often vary within a user group, and this can contribute to the relatively large standard deviations observed in the present data. Some participants may have experienced a novelty effect (40) that temporarily increased curiosity and enthusiasm, while others may have approached the system with expectations shaped by media portrayals or science-fiction imagery (22). When such expectations do not align with the functional capabilities of a real system, initial disappointment may occur.

Second, the present sample size was limited due to operational and availability constraints and therefore provided sufficient statistical power primary for the detection of large effects. As a result, smaller or moderate associations between acceptance dimensions may have remains undetected. Consequently, non-significant findings should not be interpreted as evidence for the absence of relationships between variables, but rather as indicative of the exploratory character of the study.

Third, although the internal consistency of most adapted TUI scales was acceptable, some limitations regarding construct validity should be acknowledged. The dimension “Work Routine” combined functional and affective aspects of exoskeleton use, which may limit its unidimensional interpretation despite satisfactory reliability estimates. In addition, one item within the “Usefulness” scale showed a relatively low corrected item-total correlation, indicating potential heterogeneity in how participants interpreted specific aspects of perceived usefulness. Moreover, the assessment of social perception was based on an additional single-item measure, which limits its reliability and restricts the depth of analytical interpretation. While the item provides an initial indication of appearance-related or stigma-related aspects of exoskeleton use, it does not constitute a fully validated multi-item construct. These findings suggest that while the adapted scales provide an overall reliable assessment of acceptance-related constructs, their validity should be considered exploratory and further refined in future studies.

Fourth, the study involved a restricted range of tasks that, while realistic, did not comprise the full variety of activities in which a shoulder exoskeleton might be used. In particular, tasks involving prolonged overhead work or complex multi-joint coordination were not entirely examined, which may have influenced perceptions of support and usefulness.

Fifth, the remaining TUI dimensions (e.g., curiosity, interest, technology anxiety, accessibility, and immersion) were not included, as they were not considered central to the evaluation objectives of this study. However, these dimensions can be valuable in future investigations, for example to explore long-term attitudes towards wearable support systems or to assess technology-related dispositions independent of specific applications.

## 5 Conclusion

This field study provides first insights into how an active shoulder exoskeleton for CBRN scenarios is perceived during real operational tasks in a military setting. Participants generally evaluated the device as easy to use and reasonably comfortable, yet the perceived level of mechanical support and functional usefulness remained moderate. The considerable interindividual variability, particularly in perceived usefulness, impact on work routine, and intention to use, highlights the heterogeneous nature of user experiences.

Although previous laboratory research on the same device has demonstrated clear biomechanical benefits, the present findings underline that such objective improvements are not necessarily recognized during short-term exposure in complex field conditions. Instead, users’ perceptions appear strongly shaped by the noticeability of support, the relevance of assistance for specific tasks, and the perceived effort–benefit ratio of donning the system. Moreover, participants expressed a desire for broader functional applicability, suggesting that future exoskeletons may require more versatile or multi-joint support concepts to increase perceived value.

## Data Availability

The original contributions presented in the study are included in the article/supplementary material, further inquiries can be directed to the corresponding author.

## Appendix 1

**Table A1:** Item means, standard deviations and corrected item-total correlations.

Items	N	Mean (SD)	Corrected item total correlation
Intention to use			
Item 1	27	3.33 (2.13)	0.87
Item 2	27	3.56 (2.26)	0.90
Item 3	27	3.37 (2.17)	0.91
Usability			
Item 4	27	5.22 (1.71)	0.76
Item 5	27	3.85 (1.73)	0.59
Item 6	27	6.37 (1.31)	0.47
Usefulness			
Item 7	27	3.38 (1.98)	0.34
Item 8	27	3.58 (2.06)	0.75
Item 9	27	2.46 (2.19)	0.74
Item 10	27	2.77 (1.90)	0.77
Skepticism			
Item 11	27	2.15 (1.46)	0.61
Item 12	27	1.44 (0.93)	0.55
Item 13	27	2.96 (1.37)	0.65
Item 14	27	2.52 (1.67)	0.61
Work routine			
Item 15	27	3.59 (2.12)	0.77
Item 16	27	2.78 (1.67)	0.44
Item 17	27	2.96 (1.99)	0.74
Wearing comfort			
Item 18	27	4.59 (1.85)	0.44
Item 19	27	5.30 (1.59)	0.48
Item 20	27	4.04 (1.53)	0.63
